# Gamma Irradiation-Inactivated Respiratory Syncytial Virus Vaccine Provides Protection but Exacerbates Pulmonary Inflammation by Switching from Prefusion to Postfusion F Protein

**DOI:** 10.1128/spectrum.01358-23

**Published:** 2023-06-05

**Authors:** Fengjia Chen, Hae-Ran Park, Hyun Jung Ji, Yeongkag Kwon, Min-Kyu Kim, Joon Young Song, Ki Bum Ahn, Ho Seong Seo

**Affiliations:** a Accelerator Radioisotope Research Section, Advanced Radiation Technology Institute, Korea Atomic Energy Research Institute, Jeongeup, Republic of Korea; b Department of Oral Microbiology and Immunology, DRI, and BK21 Plus Program, School of Dentistry, Seoul National University, Seoul, Republic of Korea; c Department of Internal Medicine, Korea University College of Medicine, Seoul, Republic of Korea; d Animal Production and Health Laboratory, Joint FAO/IAEA Centre for Nuclear Applications in Food and Agriculture, Department of Nuclear Sciences and Applications, International Atomic Energy Agency, Seibersdorf, Austria; e Department of Radiation Science, University of Science and Technology, Daejeon, Republic of Korea; Chinese Academy of Sciences Wuhan Institute of Virology

**Keywords:** respiratory syncytial virus, enhanced respiratory disease, inactivated vaccine, gamma irradiation inactivation, prefusion F, postfusion F

## Abstract

Respiratory syncytial virus (RSV) is a common respiratory pathogen that causes lower respiratory diseases among infants and elderly people. Moreover, formalin-inactivated RSV (FI-RSV) vaccine induces serious enhanced respiratory disease (ERD). Radiation has been investigated as an alternative approach for producing inactivated or live-attenuated vaccines, which enhance the antigenicity and heterogeneous protective effects of vaccines compared with conventional formalin inactivation. In this study, we developed an RSV vaccine using gamma irradiation and analyzed its efficacy against RSV vaccine-induced ERD in a mouse model. Although gamma irradiation-inactivated RSV (RI-RSV) carbonylation was lower than FI-RSV carbonylation and RI-RSV showed a significant antibody production and viral clearance, RI-RSV caused more obvious body weight loss, pulmonary eosinophil infiltration, and pulmonary mucus secretion. Further, the conversion of prefusion F (pre-F) to postfusion F (post-F) was significant for both RI-RSV and FI-RSV, while that of RI-RSV was significantly higher than that of FI-RSV. We found that the conversion from pre- to post-F during radiation was caused by radiation-induced reactive oxygen species. Although we could not propose an effective RSV vaccine manufacturing method, we found that ERD was induced by RSV vaccine by various biochemical effects that affect antigen modification during RSV vaccine manufacturing, rather than simply by the combination of formalin and alum. Therefore, these biochemical actions should be considered in future developments of RSV vaccine.

**IMPORTANCE** Radiation inactivation for viral vaccine production has been known to elicit a better immune response than other inactivation methods due to less surface protein damage. However, we found in this study that radiation-inactivated RSV (RI-RSV) vaccine induced a level of immune response similar to that induced by formalin-inactivated RSV (FI-RSV). Although RI-RSV vaccine showed less carbonylation than FI-RSV, it induced more conformational changes from pre-F to post-F due to the gamma radiation-induced reactive oxygen species response, which may be a key factor in RI-RSV-induced ERD. Therefore, ERD induced by RSV vaccine may be due to pre-F to post-F denaturation by random protein modifications caused by external stress. Our findings provide new ideas for inactivated vaccines for RSV and other viruses and confirm the importance of pre-F in RSV vaccines.

## INTRODUCTION

Respiratory syncytial virus (RSV), a member of the family *Paramyxoviridae*, consists of a group of negative-sense single-stranded RNA viruses that were first discovered among a colony of chimpanzees with coryza in 1956 (1). RSV causes lower respiratory disease in infants and toddlers worldwide and is identified as a significant problem among the elderly (2–6). RSV is the most common cause of acute bronchiolitis and pneumonia (7, 8). Inflammation of the small airways after RSV infection causes necrosis of lung cells, and their debris causes ball-valve airway obstruction, resulting in pulmonary hyperinflation in the distal airways (7). RSV causes approximately 3.2 million hospitalizations and 66,000 to 234,000 deaths worldwide annually among children aged under 6 months (9). Vaccination is the most effective way of preventing RSV infection; however, the formalin-inactivated RSV (FI-RSV) vaccine tested among infants and children in the United States in 1966 resulted in a significant increase in the frequency of lower respiratory tract illness upon natural RSV infection (10, 11). Eighty percent of FI-RSV vaccine recipients were hospitalized, and two vaccinated infants died from subsequent RSV infection (12). Autopsies performed on the lungs of the children who died of FI-RSV revealed bronchopneumonia with extensive monocyte infiltration and pulmonary eosinophilia, typical of enhanced respiratory disease (ERD) (1, 8).

Several attempts have been made to elucidate the pathogenesis of FI-RSV vaccine-induced ERD. FI-RSV can cause severe ERD, including weight loss, eosinophilia, and lung histopathology, in animal models (13). The Th2-biased immune response to RSV infection is significantly involved in ERD pathogenesis (14). The two major glycoproteins on the surface of RSV, the fusion (F) and attachment (G) glycoproteins, control the early phase of RSV infection and pathogenesis (15). Recent studies have shown that RSV-neutralizing antibodies recognize epitopes found exclusively in the prefusion form of the F glycoprotein (pre-F) (16, 17). Therefore, vaccines should induce high levels of neutralizing antibodies directed against pre-F without predisposing patients to ERD.

Clinical trials of several types of RSV vaccine candidates endorsing pre-F as a new target are ongoing among pregnant women and the elderly, and at least four of these candidates are in phase 3 trials (18). Pfizer’s bivalent pre-F recombinant vaccine (RSVPreF) showed 85.7% efficacy in older-adult trials (NCT05035212) and 81.8% and 69.4% efficacy in maternal trials among 90-day-old and 6-month-old infants, respectively (NCT04424316) (19, 20). In the GSK RSV older-adult vaccine candidate (RSVPreF3) phase 3 trial, AS01E-adjuvanted pre-F subunit vaccine demonstrated 82.6% overall efficacy and 94.1% efficacy against severe disease (21). The Ad26.RSV.pre-F vaccine candidate produced by Johnson & Johnson, which expresses the pre-F protein through an adenoviral vector, is being developed for the elderly (22). Despite the success of the mRNA vaccine against COVID-19, Moderna has received FDA approval for the mRNA-1345 vaccine, which encodes stabilized RSV pre-F, a fast-track license to fight RSV infections among people aged over 60 years (23). In addition to the four pre-F-dependent vaccine candidates mentioned above, the Bavarian Nordic vaccine candidate (MVA-BN-RSV), a poxvirus vector expressing RSV antigens F, G, M2, and N, is the only non-pre-F-dependent phase 3 trial vaccine (18). Moreover, clinical studies and developments of various types of vaccines, such as live RSV vaccines (RSV-MinL4.0, VAD0001, and MV-012-968), RSV F nanoparticles, and virus-like particles (VLPs), are ongoing (18). However, the most important issue to consider in the development of such a vaccine is whether it induces ERD.

A previous study indicated that FI-RSV induces ERD by carbonylation of viral antigens (14). Therefore, alternative inactivation methods have been introduced in preclinical studies. For example, chemically inactivated (2,2-dithiodipyridine zinc finger-reactive compound inactivated) RSV vaccines supplemented with a Ribi adjuvant system (RAS) showed significant immunogenicity but induced ERD (24). A beta-propiolactone (BPL)-inactivated RSV vaccine enhanced RSV-specific immunity and reduced production of mRNA of lung eotaxin, a chemokine associated with eosinophil influx (25). The efficacy of UV-inactivated (UV-RSV) vaccines is low, which is due to the low affinity of RSV-specific antibodies induced compared with live RSV (26). Owing to its simple manufacturing process, cost effectiveness, lack of chemicals, and superior antigenicity, gamma irradiation has been investigated as an alternative to the developed inactivated vaccines (27). Gamma irradiation induces relatively low carbonylation, even at high doses. These advantages suggest that gamma irradiation may be useful for the development of inactivated RSV vaccines that do not cause ERD. A corresponding report showed that the low-energy electron irradiation (LEEI)-RSV vaccine produces only an extremely small amount of X rays as a by-product, causing a strong immune response that results in a drastic drop in the viral load without enhancing weight loss or hair disorganization. However, the study did not determine whether the LEEI-RSV vaccine induced ERD in the mouse model (28).

In the present study, we investigated whether a gamma irradiation-inactivated RSV (RI-RSV)-induced immune response could lead to ERD by restimulation with live RSV. However, we found that RI-RSV caused more severe ERD than did FI-RSV and that these immune responses are likely due to pre-F to post-F conformational changes by radiation-induced redox oxygen species (ROS). Therefore, FI-RSV-induced ERD is not attributed to carbonylation but is more likely to be a pre-F to post-F conformational change caused by extracellular chemical reactions.

## RESULTS

### Preparation of gamma irradiation-inactivated RSV vaccine with reduced carbonylation.

**FIG 1:**
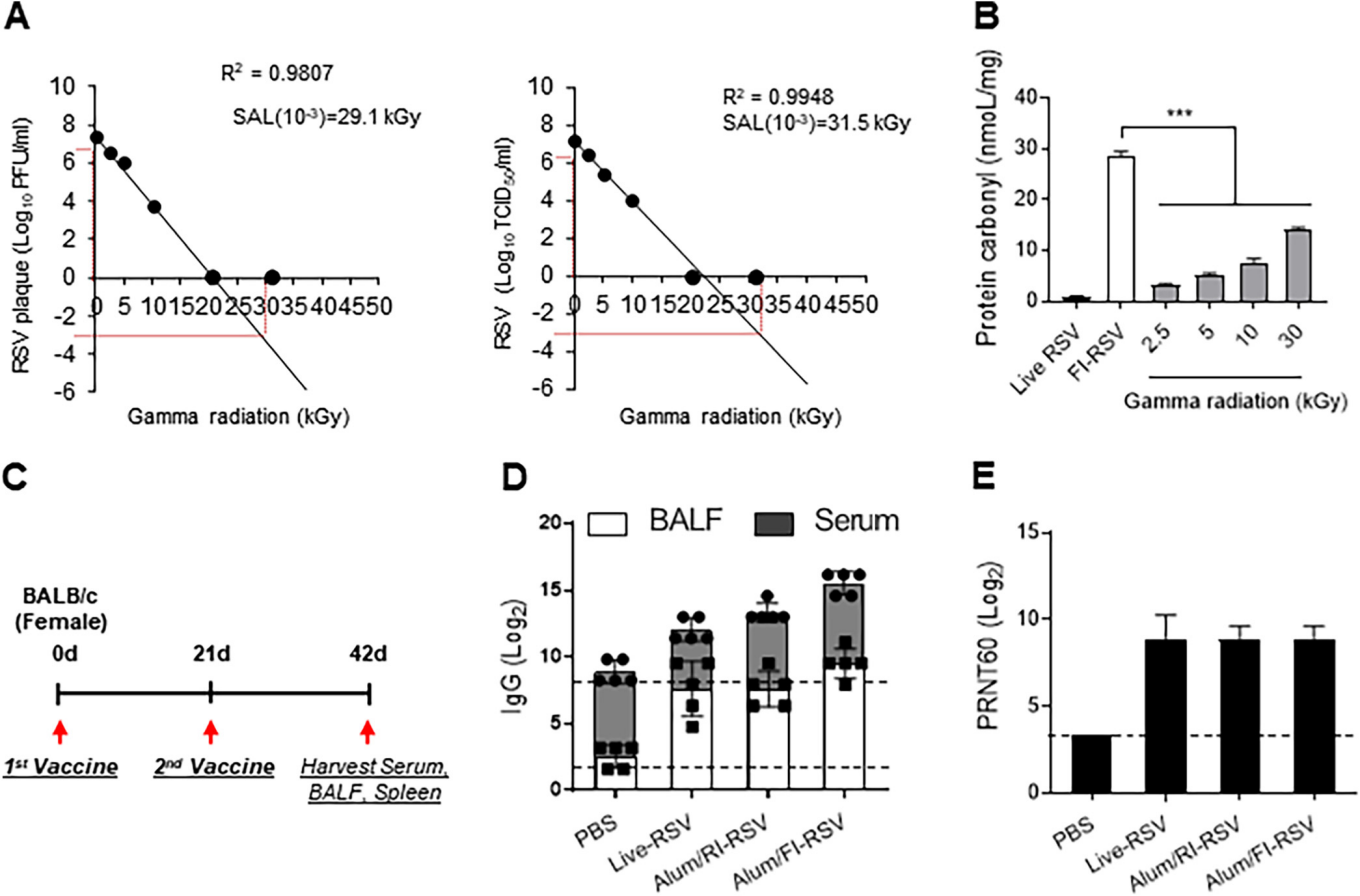
Gamma irradiation-inactivated RSV vaccine and its immunogenicity. (A and B) Gamma irradiation-inactivated RSV vaccine production. Live RSV (2 × 10^6^ PFU/mL) was irradiated with 0-, 2.5-, 5-, 10-, 20-, and 30-kGy gamma rays, and viral viability was calculated by plaque assay (left panel) and TCID_50_ assay (right panel) (A). Protein carbonylation of live RSV, RI-RSV, and FI-RSV was measured by DNPH immunoassay (B). (C to E) Immunogenicity of live RSV, FI-RSV, and RI-RSV vaccines. Experimental vaccination scheme. BALB/c mice (*n* = 5 per group) were immunized twice intramuscularly with 100 μL of live-RSV, RI-RSV or FI-RSV (5 × 10^5^ PFU) absorbed with 100 μL of aluminum hydroxide. Three weeks after the last immunization (day 42), mouse serum, BALF and spleen were collected (C). RSV-specific IgG levels in sera or BALF were assessed using RSV (2 × 10^5^ PFU/mL, 100 μL)-immobilized ELISA plates. Endpoints were 1.58 for BALF and 8.23 for serum (D). RSV neutralization antibody titers were calculated by plaque reduction neutralization assay (E). Data are presented as mean values ± SD of triplicate samples. The asterisks indicate significant differences compared with the FI-RSV group (***, *P < *0.001).

Gamma irradiation inactivation methods have been reported to reduce protein carbonylation in bacterial vaccines (29). To determine whether radiation promotes less carbonylation, which affects the inactivated RSV vaccine-induced ERD, an inactivated RSV vaccine was prepared using gamma irradiation. Gamma irradiation-inactivated RSV vaccine was prepared according to the sterility assurance level (SAL) used in the manufacture of virus vaccines (30, 31). Live RSV (2 × 10^6^ PFU/mL) was irradiated with gamma-radiation (Co^60^) for 2 h at 2.5, 5, 10, 20, and 30 kGy, and the viability of RSV was determined using plaque or 50% tissue culture infective dose (TCID_50_) assay (Fig. 1A). The SAL value (−10^3^) was calculated as assessed by linear regression performed with PFU or TCID_50_. Although we did not detect live RSV with gamma irradiation above 20 kGy, the SAL value indicated that the optimal dose to inactivate live RSV ranged from 29.1 to 31.5 kGy. Therefore, we set the RSV inactivation radiation dose to 30 kGy. To confirm that the carbonylation rate was significantly reduced compared to that with the formalin-inactivated method, RSV proteins were extracted from irradiated or formalin-inactivated RSV and the ratio of carbonylated proteins was measured using a 2,4-dinitrophenylhydrazine (DNPH) immunoassay. As shown in Fig. 1B, gamma irradiation-inactivated RSV showed significantly lower levels of protein carbonylation (13.98 ± 0.58 nmol/mL at 30 kGy) than formalin-inactivated RSV (28.71 ± 0.75 nmol/mL).

To examine the immunogenicity of RI-RSV vaccine compared to live RSV and FI-RSV, BALB/c mice (*n* = 5) were immunized intramuscularly with 5 × 10^5^ PFU of each vaccine emulsified with aluminum hydroxide adjuvant on days 0 and 21. At day 21 after the second vaccination (day 42), mouse bronchoalveolar lavage fluid (BALF) and serum were collected (Fig. 1C), and RSV-specific and RSV-neutralizing antibodies were measured. As shown in Fig. 1D, RSV-specific IgG levels in the serum and BALF were significantly increased in all RSV groups compared to the phosphate-buffered saline (PBS)-immunized group, but there was no significant difference among the vaccinated groups. Subsequently, a 60% plaque reduction neutralizing titer (PRNT_60_) assay was performed to compare the RSV-neutralizing activity of mouse BALF (Fig. 1E). Similar PRNT_60_s were observed among the vaccinated groups. As expected, the immunogenicity of the radiation vaccine recipe was similar to that of FI-RSV, but it induced less protein carbonylation.

### Lightly carbonylated RI-RSV also caused ERD *in vivo*.

**FIG 2:**
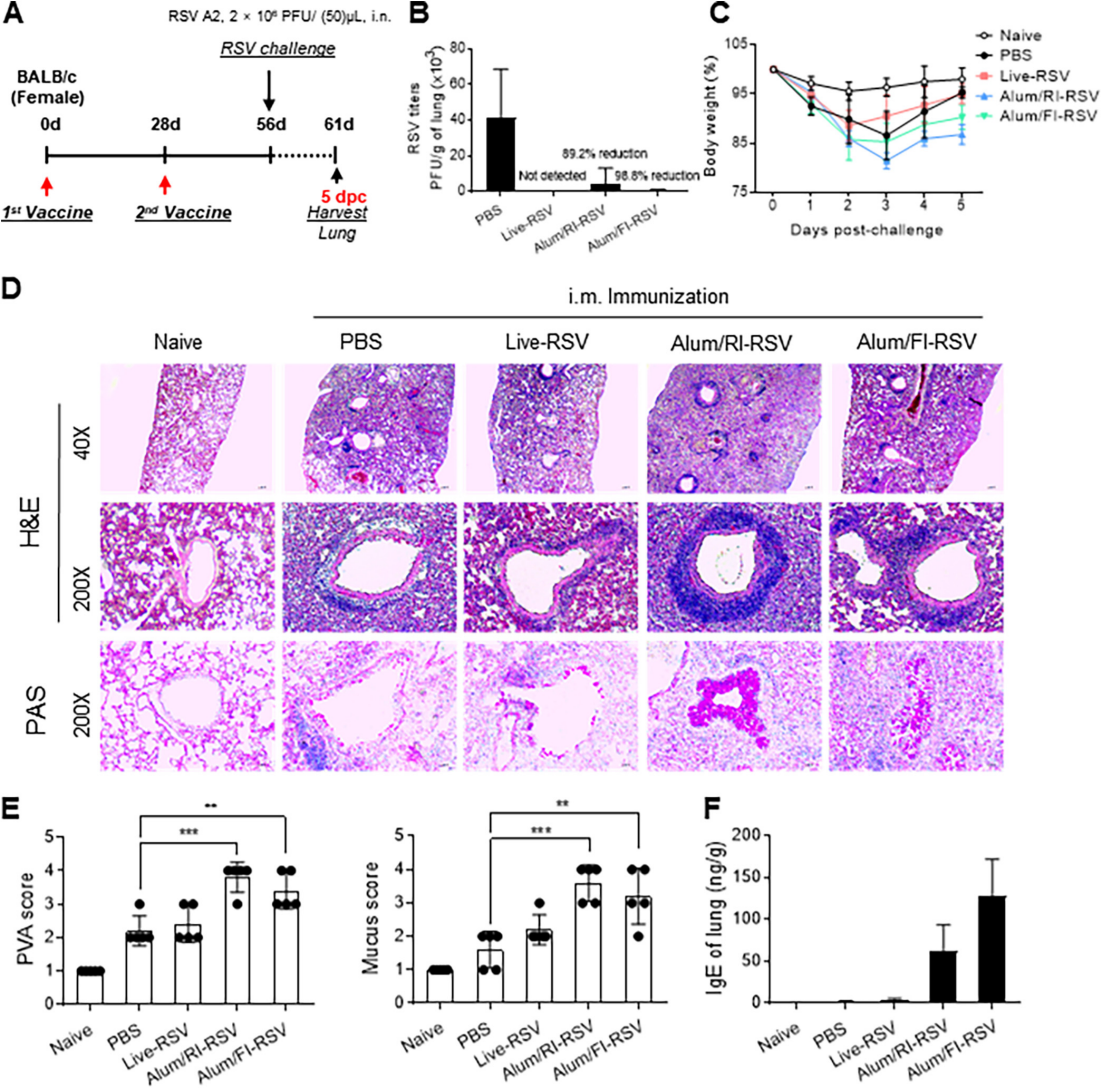
Lightly carbonylated RI-RSV induced severe ERD *in vivo*. (A) Experimental vaccination and challenge scheme. BALB/c mice (*n* = 5 per group) were immunized intramuscularly twice at a 4-week interval with RI-RSV (5 × 10^5^ PFU) or FI-RSV (5 × 10^5^ PFU) with alum (20 μg) adjuvant or intranasally (i.n.) with live RSV (5 × 10^5^ PFU). At 4 weeks after the second immunization, the mice were challenged i.n. with live RSV A2 strain (2 × 10^6^ PFU). (B and C) Protective response by vaccination. Mouse lungs were homogenized with 2-mm beads at 5 dpc (day 61), and RSV titers were measured from the supernatant using plaque assay (B) and mouse body weight was monitored daily for 5 days from the time of the RSV challenge (day 56) (C). (D and E) Histopathological changes in the lung tissue infected with live RSV at 5 dpc (day 61). Representative histological images of H&E- or PAS-stained lung tissue sections are shown (D). The perivascular aggregates of leukocytes (PVA) and mucus secretion scores were calculated (E). (F) The level of IgE in the supernatant of lung homogenates was measured by ELISA. Data are presented as mean values ± SD of five samples. The asterisks indicate significant differences compared with the PBS-immunized groups (**, *P < *0.01; ***, *P < *0.001).

Heavily carbonylated FI-RSV is the key modification that causes ERD in clinical trials and serves as a significant obstacle in the development of novel RSV vaccines (14). Therefore, we examined whether the more lightly carbonylated RI-RSV vaccine led to reduced ERD compared to that with FI-RSV. BALB/c mice were immunized twice with live RSV only, FI-RSV with alum, or RI-RSV with alum and then challenged with live RSV (2 × 10^6^ PFU/mouse) on day 56. At 5 days postchallenge (dpc; day 61), ERD was analyzed by measuring the lung histopathology and IgE production in the lungs (Fig. 2A). As shown in Fig. 2B, pulmonary RSV titers decreased drastically in all vaccinated groups compared to those in the unvaccinated group, indicating that immune responses induced by live RSV, FI-RSV, or RI-RSV vaccination successfully prevented RSV generation and/or transmission in the lungs. However, despite the decrease in pulmonary RSV titers, body weight did not improve in any of the vaccinated groups. Notably, the groups vaccinated with RI-RSV and FI-RSV showed a slightly more severe (but not significant) trend than the unvaccinated group (Fig. 2C).

To determine whether the severity of ERD in vaccinated mice was due to RSV vaccine-induced ERD, mouse lung histopathology was analyzed on day 61 (Fig. 2D). Infiltration of immune cells and mucus-producing cells into the lung tissue was visualized by hematoxylin and eosin (H&E) and periodic acid-Schiff (PAS) staining, and severity was scored using perivascular aggregates of leukocytes (PVA) and mucus scores (Fig. 2E). After 5 dpc (day 61), we found a modest infiltration of immune cells into the lungs in both the PBS- and live RSV-immunized groups, and few parabronchial mucus sections were detected. In contrast, significant infiltration of immune and mucus-producing cells was observed in FI-RSV- and RI-RSV-immunized groups. Furthermore, the levels of IgE in BALF were not elevated in the PBS- or live RSV-immunized group, but a drastic increase in IgE production was observed in the RI-RSV and FI-RSV groups (Fig. 2F). These data suggest that the reduction in the body weight of RSV-vaccinated mice was due to FI-RSV- or RI-RSV-induced ERD. Contrary to previous reports (29), lightly carbonylated RI-RSV caused slightly more severe ERD than FI-RSV as determined by analysis based on body weight and lung histopathological findings.

### RI-RSV induced Th2-biased immune responses.

**FIG 3:**
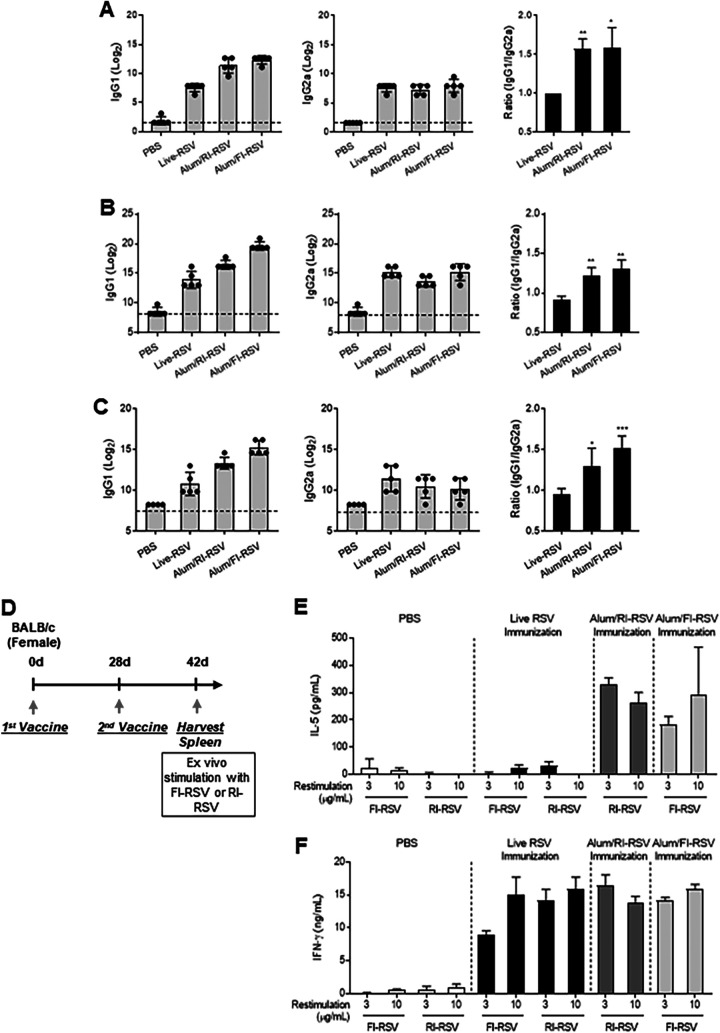
Th2-biased immune responses by FI- or RI-RSV immunization. (A to C) Production of RSV-specific IgG1 and IgG2a in the BALF (A) and sera (B) and the calculated IgG1/IgG2a ratios. Prefusion F protein-specific IgG1 and IgG2a levels in sera from immunized mice were assessed using prefusion F protein (300 ng/mL, 100 μL, DS-Cav1)-immobilized ELISA plates. The production of prefusion F protein-specific IgG1 and IgG2a in the sera was detected, and IgG1/IgG2a ratio was calculated (C). Endpoints were 1.58 for BALF and 8.23 for serum. (D to F) Th1 and Th2 cytokine production by splenocytes. BALB/c mice (*n* = 5 per group) were immunized twice intramuscularly with 100 μL of live RSV, RI-RSV, or FI-RSV (5 × 10^5^ PFU) absorbed with 100 μL of aluminum hydroxide. Three weeks after the last immunization (days 42), mouse splenocytes were collected and stimulated with FI-RSV or RI-RSV (D). Splenocytes from immunized mice were stimulated with RI-RSV (1 × 10^5^ or 3 × 10^5^ PFU/mL equivalent with 1 or 3 μg of protein) or FI-RSV (1 × 10^5^ or 3 × 10^5^ PFU/mL) for 3 days, followed by examination of the levels of IL-5 (E) and IFN-γ (F) production in the culture supernatants. Data are presented as mean values ± SD of five samples. The asterisks indicate significant differences compared with the live-RSV-immunized groups (*, *P < *0.05; **, *P < *0.01; ***, *P < *0.001).

Th2-biased immune responses are the major cause of ERD (12, 32). To investigate whether RI-RSV-induced ERD was due to a Th2-biased immune response, such as FI-RSV, the levels of anti-RSV IgG1 (Th2-type antibody) and anti-RSV IgG2a (Th1-type antibody) were measured in the BALF (Fig. 3A) and sera (Fig. 3B). The live-RSV group showed similar levels of IgG1 and IgG2a, at a ratio of approximately 1. In contrast, both the RI-RSV and FI-RSV groups showed higher IgG1 levels, but similar levels of IgG2a were observed in the live-RSV group. Both the RI-RSV and FI-RSV groups showed significantly higher IgG1/IgG2a ratios than did the live-RSV group: for BALF, the values were 1.59 ± 0.26 and 1.57 ± 0.13 for FI-RSV and RI-RSV, respectively, and for sera, the values were 1.30 ± 0.12 and 1.21 ± 0.11 for FI-RSV and RI-RSV, respectively. In addition, we measured the levels of IgG1 and IgG2a against RSV prefusion F protein (DS-Cav1) in the sera. As shown in Fig. 3C, we observed higher IgG1/IgG2a ratios in the RI-RSV and FI-RSV groups than in the live-RSV group, consistent with the findings in Fig. 3A and B. These results suggest that both RI-RSV and FI-RSV vaccines significantly induced a Th2-biased immune response. To confirm a Th2-biased immune response by RI-RSV, splenocyte interleukin 5 (IL-5; Th2) and gamma interferon (IFN-γ; Th1) cytokine production was measured after restimulation with RI-RSV or FI-RSV (Fig. 3D). When splenocytes were stimulated with FI-RSV or RI-RSV, IFN-γ was significantly secreted in all vaccinated groups (live RSV, FI-RSV, and RI-RSV); however, IL-5 was detected only in the FI- and RI-RSV groups (Fig. 3E and F). Therefore, the lower Th2 response of the live-RSV group by RI-RSV or FI-RSV restimulation indicated that live-RSV proteins lowered the Th2-related T cell immune response, and higher Th2 responses by RI-RSV or FI-RSV were likely due to alterations in RSV protein structure by formalin-induced carbonylation or gamma irradiation-induced modification.

To further confirm that the Th2-biased immune response by RI-RSV is the main cause of ERD, the well-known Th1-inducing adjuvant monophosphoryl lipid A (MPL) was applied to the RI-RSV to compare with alum adjuvant. As shown in Fig. S1A to C in the supplemental material, the IgG1/IgG2a and IL-5/IFN-γ ratios of MPL/RI-RSV group were significantly lower than those of alum/RI-RSV group. A similar neutralization effect of sera was observed in the alum/RI-RSV and MPL/RI-RSV groups (Fig. S1D and E). However, MPL/RI-RSV still induced severe weight loss and pulmonary inflammation not significantly different from that with alum/RI-RSV (Fig. S1F and G). Hence, Th2-biased responses of RI-RSV may not play a decisive role in ERD induced by RI-RSV.

### Oxidative stress induces the conversion of prefusion F protein to postfusion F protein in RSV.

**FIG 4:**
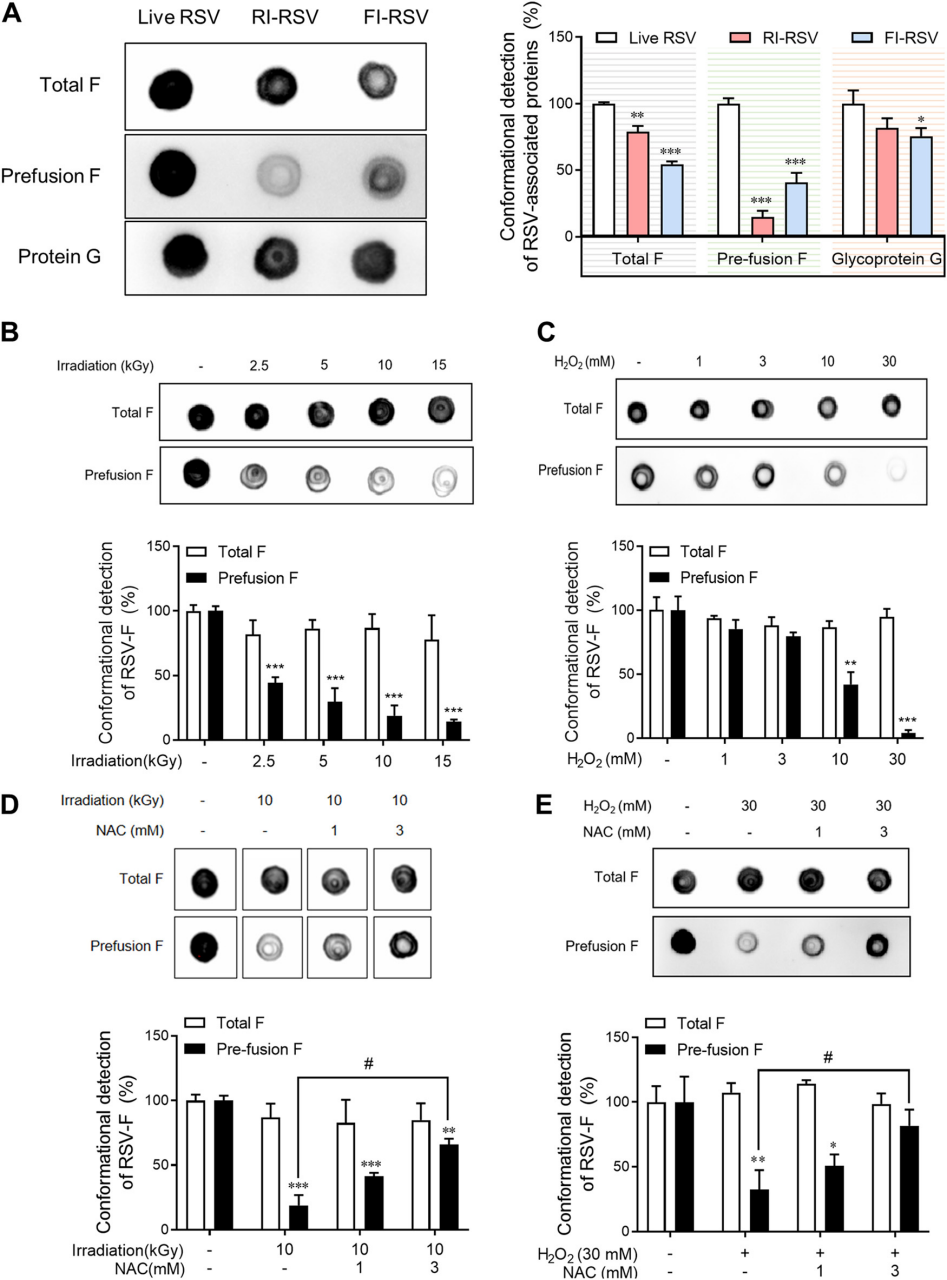
Oxidative stress induces the conversion of prefusion F protein to postfusion F protein in RSV. (A) Live RSV (5 × 10^7^ PFU/mL), RI-RSV (5 × 10^7^ PFU/mL), and FI-RSV (5 × 10^7^ PFU/mL) were prepared. (B) RSV (5 × 10^7^ PFU/mL) was gamma irradiated with 0, 2.5, 5, 10, or 15 kGy. (C) RSV (5 × 10^7^ PFU/mL) was treated with 0, 1, 3, 10, or 30 mM H_2_O_2_. (D) RSV (5 × 10^7^ PFU/mL) was gamma irradiated with 10 kGy in the presence or absence of NAC (0, 3, or 10 mM). (E) RSV (5 × 10^7^ PFU/mL) was treated with 30 mM H_2_O_2_ in the presence or absence of NAC (0, 3, or 10 mM). Samples were blotted onto a nitrocellulose membrane and detected by prefusion F protein- or total F protein-IgG, followed by addition of HRP-conjugated secondary antibodies and visualization with chemiluminescence. One of three similar results is shown in the top portion. In the bottom portion, the densities of proteins, as quantified by densitometry analysis using ImageJ software, are shown. Data are presented as mean values ± SD of triplicate samples. The asterisks indicate significant differences compared with live RSV (*, *P < *0.05; **, *P < *0.01; ***, *P < *0.001). *#*, *P < *0.05 compared to gamma irradiation or H_2_O_2_ treatment group.

The RSV fusion (F) glycoprotein undergoes drastic conformational change from a prefusion to a postfusion state during viral entry (33). The post-F conformation exhibits fewer potential neutralizing epitopes and less fusion-inhibitory activity than the pre-F conformation, which may be a key trigger for the ERD state (14, 34). To investigate whether gamma irradiation inactivation affects F protein conformational changes, the amounts of pre-F protein were detected using a dot blot assay. As shown in Fig. 4A, the level of pre-F (anti-prefusion conformation of HRSV F protein [RSB20]) protein was sharply reduced after radiation or formalin inactivation, but the amounts of total G protein and total F protein (anti-prefusion and postfusion conformation of HRSV F protein [RSB15]) did not change. The right side of Fig. 1A shows the normalized amounts of total F protein and prefusion F protein by RSV G protein, and the tendency is consistent with the above-mentioned results. These results suggest that a conformational change occurred from the pre-F protein to the post-F protein, which did not interact with the pre-F-specific antibody.

Subsequently, to verify whether radiation was the main cause of the conversion, the conformational change in the prefusion F protein induced by gamma irradiation was examined at various doses. The detection of prefusion F protein was reduced from 2.5 kGy, and the reduction effect increased in a dose-dependent manner up to 15 kGy of gamma irradiation; however, there was no significant change in the levels of total F protein (Fig. 4B).

Ionizing radiation elevates the production of ROS through water radiolysis, which damages cellular constituents such as nucleic acids or proteins (27). To clarify whether this deformation of the prefusion F protein was due to oxidative stress, the conformational change in the prefusion F protein by gamma irradiation was detected at various doses of an oxidizing agent, hydrogen peroxide (H_2_O_2_; 0, 1, 3, 10, and 30 mM) or in the presence of an antioxidant, *N*-acetylcysteine (NAC; 0, 1, or 3 mM). Consistent with the irradiation results, the reduction in prefusion F increased in a dose-dependent manner after treatment with H_2_O_2_ (Fig. 4C). As expected, the reduction in the level of prefusion F protein induced by gamma irradiation or H_2_O_2_ was reversed by NAC treatment in a dose-dependent manner (Fig. 4D and E). These results indicate that the conversion of prefusion F to postfusion F by gamma irradiation may be due to the oxidative stress caused by ROS. Therefore, RSV vaccine development through inactivation by gamma irradiation or ROS, such as hydrogen peroxide, induces low carbonylation, unlike formalin, but induces another protein modification by ROS. Therefore, it is not an effective RSV vaccine development method.

## DISCUSSION

RSV is a common respiratory virus that causes lower respiratory diseases, such as bronchiolitis and pneumonia, in infants and toddlers worldwide. There have been numerous attempts to develop inactivated vaccines against RSV infection, but due to the difficulty of overcoming ERD, there is currently no licensed and commercially available inactivated RSV vaccine. A recent report suggested that ERD induced by FI-RSV is likely due to formalin-induced carbonylation at the lysine residues of RSV surface proteins (14). Formalin-induced carbonylation attenuates the toxins and produces toxoid vaccines (35). Although the indirect effects of gamma irradiation-induced ROS can cause similar modifications to proteins and lipids, previous reports and the present study have shown that the RI of viruses or bacteria induces lower carbonylation than FI (29). However, RI-RSV induced more severe ERD than FI-RSV, despite inducing a small amount of carbonylation. Therefore, protein carbonylation may not be the key to RSV-induced ERD and other types of gamma irradiation-specific protein modifications may induce ERD.

The RSV envelope contains three viral transmembrane surface glycoproteins (glycoprotein G, fusion protein F, and small hydrophobic SH proteins) and a nonglycosylated matrix (M) protein (36–38). However, the protein(s) involved in FI-RSV-induced ERD is unclear. Among the RSV proteins, the F protein, which presents conformations of prefusion (pre-F) and postfusion (post-F) before and after virus-cell interaction, is considered the most important antigen of RSV and is used for vaccine development (39, 40). High-titer neutralizing antibodies against RSV isolated from natural RSV-infected individuals mainly recognized the prefusion conformational epitopes; some only recognized the prefusion protein structure and not the postfusion protein structure, such as the D25 antibody, which recognized only the epitope φ available in the prefusion structure (38). Studies show that formalin can convert pre-F of RSV to post-F and that post-F adjuvanted with GLA-SE and alum induce pulmonary inflammation in cotton rats (34). In contrast, numerous studies have shown that pre-F can provide powerful vaccine efficacy and does not induce ERD (18). Many subunit vaccines have therefore been developed using the pre-F protein. Therefore, numerous researchers have argued that pre- and postmodification of the F protein and the induction of post-F antibodies may cause ERD. In this study, we demonstrate for the first time that the conversion of pre-F to post-F proteins occurs under both formalin treatment and gamma irradiation. This protein conversion did not correlate with the carbonylation ratio. Given that RI-RSV, which exhibited a higher post-F protein conversion and lower carbonylation ratio than FI-RSV, induced more severe ERD, we concluded that the structural changes in pre-F proteins induced by formalin and gamma irradiation are more responsible for ERD development.

The FI-RSV-induced Th2 response is another immunological mechanism underlying RSV-induced ERD. FI-RSV vaccinated with alum, a Th2-biased adjuvant, induces Th2 cytokine and IL-4 production in the lungs (12). A lack of CD4^+^ T cells or double depletion of IL-4 and IL-10 reduced the severity of ERD in the lungs and increased levels of IL-5, IL-13, and Th2 cytokines were observed in mice with ERD (41, 42). Consistent with previous reports, we found that both RI-RSV and FI-RSV adjuvanted with alum induced a Th2-biased immune response. To rescue the Th2-biased immune response induced by RI-RSV, RI-RSV was adjuvanted with MPL, a well-known Th1-inducing adjuvant. MPL/RI-RSV reduced the IgG1/IgG2a and IL-5/IFN-γ ratios but still caused more severe weight loss and pulmonary inflammation (Fig. S1). Therefore, our results suggest that Th2-biased responses do not play a crucial role in RI-RSV-induced ERD; additional mechanistic studies are needed to elucidate RSV-induced ERD.

Gamma irradiation is useful for manufacturing various inactivated vaccines because it causes less damage to antigens than chemical inactivation and would therefore be particularly suitable for the production of RSV vaccines. However, we found that the gamma irradiation method caused less carbonylation than formalin but more or similar levels of conformational changes in the F protein, which is a critical antigen for the RSV vaccine. This conformational change from pre-F to post-F was attributed to ROS induced by the indirect effect of radiation. Therefore, ERD induced by the FI-RSV vaccine is not simply caused by formalin but might be due to the denaturation of pre-F to post-F by random protein modification by external stress. Therefore, these technical issues during the manufacturing process should be considered when developing RSV vaccines.

## MATERIALS AND METHODS

### Reagents.

Dulbecco’s modified Eagle’s medium (DMEM), red blood cell (RBC) lysis buffer, hydrogen peroxide (H_2_O_2_) solution, crystal violet, *N*-acetylcysteine (NAC), and monophosphoryl lipid A (MPL) were purchased from Sigma-Aldrich (St. Louis, MO, USA). RPMI 1640 medium and fetal bovine serum (FBS) were purchased from Biowest (Nuaillé, France). Penicillin-streptomycin solution was purchased from HyClone (Logan, UT, USA). SeaPlaque agarose was obtained from Lonza (Rockland, ME, USA). Alhydrogel adjuvant was purchased from InvivoGen (San Diego, CA, USA). A formaldehyde solution was obtained from JUNSEI (Tokyo, Japan). Enzyme-linked immunosorbent assay (ELISA) kits for IL-5 and IFN-γ were purchased from BD Biosciences (San Diego, CA, USA). An IgE ELISA core kit was purchased from Cusabio (Houston, TX, USA). Hematoxylin and eosin (H&E) staining solutions were purchased from Agilent Dako (Santa Clara, CA, USA). The periodic acid-Schiff (PAS) staining kit and anti-RSV glycoprotein antibody (8C5 [9B6]) specific for the G protein were purchased from Abcam (Cambridge, UK). Anti-prefusion F protein (RSB20) and anti-prefusion and postfusion F protein antibody (RSB15) were purchased from Absolute Antibodies (Wilton, UK). Horseradish peroxidase (HRP)-conjugated anti-mouse IgG, IgG1, and IgG2a were purchased from Southern Biotech (Birmingham, AL, USA). The protein carbonyl colorimetric assay kit was purchased from Cayman Chemical (Ann Arbor, MI, USA).

### Virus and cell culture.

The RSV A2 strain was a gift from the International Vaccine Institute (Seoul, Republic of Korea) and was propagated in human laryngeal epithelial (HEp-2) cells (CCL-23; American Type Culture Collection, Manassas, VA, USA) in 175-cm^2^ flasks. HEp-2 cells were cultured in DMEM supplemented with 10% FBS and penicillin-streptomycin solution at 37°C in a humidified incubator with 5% CO_2_.

### Preparation of live RSV, RI-RSV, and FI-RSV.

A high concentration of RSV was harvested as previously described (43), with slight modifications. First, 90% confluent HEp-2 cells were infected with seed RSV at a multiplicity of infection of 0.01 for 2 h at 37°C. The cells were then incubated for 72 h in DMEM containing 2% FBS, which was subsequently replaced with fresh FBS-free DMEM for 48 h. Infected cells were scraped and lysed by freeze-thawing three times. Following centrifugation (4,000 × *g*, 20 min, 4°C), RSV in the supernatant was aliquoted and stored at −80°C until further use. RI-RSV was prepared by gamma irradiation at different intensities in the presence or absence of antioxidants. FI-RSV was prepared as described in a previous study (43). RSV was inactivated with 0.025% formalin for 72 h at 37°C. Then FI-RSV was pelleted by ultracentrifugation (30,000 × *g*, 1 h, 4°C) to remove formaldehyde and resuspended in an FBS-free medium. RI-RSV or FI-RSV was adsorbed onto MPL (100 μg/mL) or Alhydrogel (4 mg/mL) adjuvant.

### Plaque and tissue culture infectious dose assays.

Inactivation of RI-RSV and FI-RSV was confirmed by plaque and TCID_50_ assays as described previously (44, 45), but with slight modifications. For plaque assay, HEp-2 cells (4 × 10^5^ cells/well) were seeded in 12-well plates and incubated overnight. The following day, 200 μL of serially diluted live RSV, RI-RSV, or FI-RSV was inoculated into 90 to 100% confluent cells and incubated for 2 h at 37°C. Unbound viruses were removed and 1 mL of overlay (DMEM containing 2% FBS and 1% low-melting-point agar) was added to cover the cells. Following 3 days of incubation, the cells were fixed with 4% formaldehyde and stained with 0.05% crystal violet. For the TCID_50_ assay, HEp-2 cells (2 × 10^4^ cells/well) were seeded into 96-well plates and incubated overnight. The cells were infected with 50 μL of serially diluted RSV for 2 h at 37°C, after which the medium was replaced with a fresh one containing 2% FBS, followed by incubation for 5 days. The attached cells were stained with 0.05% crystal violet and the RSV titer was calculated using the Reed and Muench calculator (46). To measure the sterility assurance level (SAL) value, 2 × 10^6^ PFU/mL of RSV was irradiated with gamma radiation (Co^60^) for 2 h at 2.5, 5, 10, 20, and 30 kGy, and the viability of RSV was determined using plaque or TCID_50_ assay. The SAL value of −10^3^ was calculated as assessed by linear regression performed with PFU or TCID_50_.

### Measurement of protein carbonylation.

Protein carbonylation of live RSV, RI-RSV, and FI-RSV was performed using a protein carbonyl colorimetric assay kit (Cayman Chemical, Ann Arbor, MI, USA) according to the manufacturer’s instructions. Summarily, 200 μL of the sample was mixed with 800 μL of 2,4-dinitrophenylhydrazine and 800 μL of 2.5 M HCl. After incubation for 1 h at room temperature, the proteins were precipitated using 20% trichloroacetic acid at 4°C for 5 min. The pellet was washed with 1 mL of a mixture of ethanol and ethyl acetate (1:1, vol/vol) and resuspended in 500 μL of guanidine hydrochloride, and the optical density at 380 nm (OD_380_) of the solution was measured.

### Immunization and viral challenge.

Animal experiments were approved by the Institutional Animal Care and Use Committee of the Korea Atomic Energy Research Institute (KAERI; IACUC-2019-03) and performed in accordance with the veterinary standards accepted by the KAERI Animal Care Center. Six- to 8-week-old female BALB/c mice (*n* = 5 per group) were immunized with RI-RSV (5 μg)/FI-RSV (5 μg) with MPL (10 μg) adjuvant or RI-RSV (5 μg)/FI-RSV (5 μg) with Alhydrogel (20 μg) by intramuscular injection and with live RSV (5 × 10^5^ PFU/mouse) by intranasal injection twice at 4-week intervals. Mice were challenged intranasally with RSV (2 × 10^6^ PFU/mouse) after 4 weeks of booster immunization. To estimate the effect of the vaccine, the body weight of the mice was measured daily for 4 or 5 days, and the lungs were collected for histological analysis (left lungs) or viral titration (right lungs) at 4 or 5 days postchallenge (dpc).

### Measurement of antibody responses, cytokine levels, and IgE production by ELISA.

The blood and bronchoalveolar lavage fluid (BALF) of the mice were harvested after 3 weeks of booster immunization. BALF was collected by infusing 0.8 mL of phosphate-buffered saline (PBS) into the lungs via the trachea using an intravenous catheter, and sera were isolated from the blood samples by centrifugation. To examine the levels of RSV-specific IgG, IgG1, and IgG2a, RSV (2 × 10^5^ PFU/mL, 100 μL) was immobilized in 96-well plates for 16 h at 4°C, followed by blocking with 5% skim milk in PBS. After washing thrice with PBS-T (0.05% Tween 20 in PBS), serially diluted sera or BALF was added to the wells and incubated for 2 h at room temperature. The plates were washed five times with PBS-T to remove unbound antibodies and horseradish peroxidase-conjugated anti-mouse IgG-, IgG1-, and IgG2a-specific antibodies were added. After 1 h of incubation and washing seven times, a 5,5′-tetramethylbenzidine (TMB) substrate solution was added to the plates, and the reaction was stopped using 2 N sulfuric acid. The absorbance was measured (450 nm) using an Epoch 2 microplate reader (BioTek, Winooski, VT, USA). Spleens from immunized mice were ground using a 40-μm cell strainer in RPMI 1640 medium containing 10% FBS, 100 U/mL of penicillin, and 100 μg/mL of streptomycin. The cells were then treated with RBC lysis buffer for 5 min at room temperature to lyse red blood cells. Splenocytes (5 × 10^6^ cells/mL) were stimulated with RI-RSV or FI-RSV (3 or 10 μg/mL each) for 3 days. The levels of the Th2 cytokine IL-5 and Th1 cytokine IFN-γ in the culture supernatants were determined using ELISA kits. The lung tissues of the mice were homogenized with 2-mm homogenizer beads at 5 dpc, and the level of IgE in the supernatant from the homogenized lung tissues was measured using an ELISA kit following the manufacturer’s instructions.

### Lung viral titration and neutralization of RSV.

The lung tissues of challenged mice were homogenized with 2-mm homogenizer beads at 4 or 5 dpc, and the viral titer in the supernatant was measured using a plaque assay. The antibody neutralization assay was performed as described previously (47), with slight modifications. Briefly, the sera were heated for 0.5 h at 56°C for inactivation. Serially diluted sera in FBS-free DMEM were mixed with RSV (500 PFU/mL), followed by incubation for 2 h at 37°C. The unneutralized virus was determined using a plaque assay, and neutralizing antibody titers were defined as those that resulted in a 60% reduction in RSV plaque formation (PRNT_60_).

### H&E staining.

The left lungs of the mice were collected and fixed with 4% formaldehyde in PBS for 24 h at room temperature. After washing with tap water for 2 h, tissues were sequentially dehydrated using 70%, 80%, 90%, 95%, and 100% ethanol. The tissues were then made transparent with xylene and embedded in paraffin. The paraffin-embedded lung tissues were sectioned at 5 μm, followed by heating for 30 min at 60°C to patch. After dewaxing and rehydration, the sections were stained with hematoxylin for 5 min and eosin for 30 s. Finally, the tissues were dehydrated, made transparent, and sealed with a mounting medium. An average of 5 to 10 lung slides were made per mouse (*n* = 5), 3 of which were randomly selected and imaged at magnifications of ×40 and ×200, and lung inflammation was scored on average. The perivascular aggregates of leukocytes (PVA) score was defined as follows: (i) few or no inflammatory cells around all vessels, (ii) mild or few inflammatory cells around certain vessels, (iii) moderate or few inflammatory cells around most vessels and a large number of inflammatory cells around certain vessels, and (iv) severe or a large number of inflammatory cells around most vessels.

### Periodic acid-Schiff staining.

Lung tissues were sectioned as described above. The samples were immersed in a periodic acid solution for 10 min, Schiff’s solution for 20 min, and hematoxylin for 5 min. Images were captured at magnifications of ×40 and ×200, and each lung was scored as an entirety. Mucus secretion was scored as follows: (i) no mucus around all airways, (ii) mild or little mucus around some airways, (iii) moderate or little mucus around some airways and large amounts of mucus around a few airways, and (iv) severe or large amounts of mucus around most airways.

### Dot blot assay.

Live or treated RSV was prepared at a dilution of 5 × 10^7^ PFU/mL. Samples of equal concentration were spotted onto nitrocellulose membranes and dried for 5 min at room temperature. The membranes were then blocked with 5% skim milk in Tris-buffered saline containing 0.05% Tween 20 (TBST) for 1 h. For detection of particular proteins, the membranes were incubated with 10 μg/mL of anti-total F, anti-prefusion F, or anti-G RSV primary antibodies for 3 h, following incubation with HRP-conjugated anti-IgG antibody for 1 h. The membranes were detected by chemiluminescence using a ChemiDoc Touch imaging system (Bio-Rad), and the relative protein expression levels were quantified using ImageJ software.

### Statistical analysis.

The mean ± standard deviation (SD) was obtained from at least triplicate samples for each treatment group, and mouse work was performed in one batch. Significant differences between two groups were determined using Student’s *t* test, and comparisons among three or more groups were analyzed using one-way analysis of variance (ANOVA).

## Supplementary Material

Reviewer comments

## References

[1] Blount RE, Jr, Morris JA, Savage RE. 1956. Recovery of cytopathogenic agent from chimpanzees with coryza. Proc Soc Exp Biol Med 92:544–549. doi:10.3181/00379727-92-22538. 13359460

[2] Shay DK, Holman RC, Newman RD, Liu LL, Stout JW, Anderson LJ. 1999. Bronchiolitis-associated hospitalizations among US children, 1980–1996. JAMA 282:1440–1446. doi:10.1001/jama.282.15.1440. 10535434

[3] Zhou H, Thompson WW, Viboud CG, Ringholz CM, Cheng PY, Steiner C, Abedi GR, Anderson LJ, Brammer L, Shay DK. 2012. Hospitalizations associated with influenza and respiratory syncytial virus in the United States, 1993–2008. Clin Infect Dis 54:1427–1436. doi:10.1093/cid/cis211. 22495079 PMC3334364

[4] Stockman LJ, Curns AT, Anderson LJ, Fischer-Langley G. 2012. Respiratory syncytial virus-associated hospitalizations among infants and young children in the United States, 1997–2006. Pediatr Infect Dis J 31:5–9. doi:10.1097/INF.0b013e31822e68e6. 21817948

[5] Jensen A, Simões EAF, Bohn Christiansen C, Graff Stensballe L. 2021. Respiratory syncytial virus and influenza hospitalizations in Danish children 2010–2016. Vaccine 39:4126–4134. doi:10.1016/j.vaccine.2021.05.097. 34116876

[6] Choi Y, Hill-Ricciuti A, Branche AR, Sieling WD, Saiman L, Walsh EE, Phillips M, Falsey AR, Finelli L. 2022. Cost determinants among adults hospitalized with respiratory syncytial virus in the United States, 2017–2019. Influenza Other Respir Viruses 16:151–158. doi:10.1111/irv.12912. 34605182 PMC8692803

[7] Wennergren G, Kristjánsson S. 2001. Relationship between respiratory syncytial virus bronchiolitis and future obstructive airway diseases. Eur Respir J 18:1044–1058. doi:10.1183/09031936.01.00254101. 11829086

[8] Prince GA, Curtis SJ, Yim KC, Porter DD. 2001. Vaccine-enhanced respiratory syncytial virus disease in cotton rats following immunization with Lot 100 or a newly prepared reference vaccine. J Gen Virol 82:2881–2888. doi:10.1099/0022-1317-82-12-2881. 11714962

[9] Shi T, McAllister DA, O’Brien KL, Simoes EAF, Madhi SA, Gessner BD, Polack FP, Balsells E, Acacio S, Aguayo C, Alassani I, Ali A, Antonio M, Awasthi S, Awori JO, Azziz-Baumgartner E, Baggett HC, Baillie VL, Balmaseda A, Barahona A, Basnet S, Bassat Q, Basualdo W, Bigogo G, Bont L, Breiman RF, Brooks WA, Broor S, Bruce N, Bruden D, Buchy P, Campbell S, Carosone-Link P, Chadha M, Chipeta J, Chou M, Clara W, Cohen C, de Cuellar E, Dang D-A, Dash-Yandag B, Deloria-Knoll M, Dherani M, Eap T, Ebruke BE, Echavarria M, de Freitas Lázaro Emediato CC, Fasce RA, Feikin DR, Feng L, RSV Global Epidemiology Network, et al. 2017. Global, regional, and national disease burden estimates of acute lower respiratory infections due to respiratory syncytial virus in young children in 2015: a systematic review and modelling study. Lancet 390:946–958. doi:10.1016/S0140-6736(17)30938-8. 28689664 PMC5592248

[10] Kim HW, Canchola JG, Brandt CD, Pyles G, Chanock RM, Jensen K, Parrott RH. 1969. Respiratory syncytial virus disease in infants despite prior administration of antigenic inactivated vaccine. Am J Epidemiol 89:422–434. doi:10.1093/oxfordjournals.aje.a120955. 4305198

[11] Kapikian AZ, Mitchell RH, Chanock RM, Shvedoff RA, Stewart CE. 1969. An epidemiologic study of altered clinical reactivity to respiratory syncytial (RS) virus infection in children previously vaccinated with an inactivated RS virus vaccine. Am J Epidemiol 89:405–421. doi:10.1093/oxfordjournals.aje.a120954. 4305197

[12] Acosta PL, Caballero MT, Polack FP. 2015. Brief history and characterization of enhanced respiratory syncytial virus disease. Clin Vaccine Immunol 23:189–195. doi:10.1128/CVI.00609-15. 26677198 PMC4783420

[13] Russell MS, Creskey M, Muralidharan A, Li C, Gao J, Chen W, Larocque L, Lavoie JR, Farnsworth A, Rosu-Myles M, Hashem AM, Yauk CL, Cao J, Van Domselaar G, Cyr T, Li X. 2019. Unveiling integrated functional pathways leading to enhanced respiratory disease associated with inactivated respiratory syncytial viral vaccine. Front Immunol 10:597. doi:10.3389/fimmu.2019.00597. 30984178 PMC6449435

[14] Moghaddam A, Olszewska W, Wang B, Tregoning JS, Helson R, Sattentau QJ, Openshaw PJ. 2006. A potential molecular mechanism for hypersensitivity caused by formalin-inactivated vaccines. Nat Med 12:905–907. doi:10.1038/nm1456. 16862151

[15] McLellan JS, Ray WC, Peeples ME. 2013. Structure and function of respiratory syncytial virus surface glycoproteins. Curr Top Microbiol Immunol 372:83–104. doi:10.1007/978-3-642-38919-1_4. 24362685 PMC4211642

[16] Magro M, Mas V, Chappell K, Vázquez M, Cano O, Luque D, Terrón MC, Melero JA, Palomo C. 2012. Neutralizing antibodies against the preactive form of respiratory syncytial virus fusion protein offer unique possibilities for clinical intervention. Proc Natl Acad Sci USA 109:3089–3094. doi:10.1073/pnas.1115941109. 22323598 PMC3286924

[17] Ngwuta JO, Chen M, Modjarrad K, Joyce MG, Kanekiyo M, Kumar A, Yassine HM, Moin SM, Killikelly AM, Chuang GY, Druz A, Georgiev IS, Rundlet EJ, Sastry M, Stewart-Jones GB, Yang Y, Zhang B, Nason MC, Capella C, Peeples ME, Ledgerwood JE, McLellan JS, Kwong PD, Graham BS. 2015. Prefusion F-specific antibodies determine the magnitude of RSV neutralizing activity in human sera. Sci Transl Med 7:309ra162. doi:10.1126/scitranslmed.aac4241. PMC4672383 26468324

[18] Mazur NI, Terstappen J, Baral R, Bardají A, Beutels P, Buchholz UJ, Cohen C, Crowe JE, Jr, Cutland CL, Eckert L, Feikin D, Fitzpatrick T, Fong Y, Graham BS, Heikkinen T, Higgins D, Hirve S, Klugman KP, Kragten-Tabatabaie L, Lemey P, Libster R, Löwensteyn Y, Mejias A, Munoz FM, Munywoki PK, Mwananyanda L, Nair H, Nunes MC, Ramilo O, Richmond P, Ruckwardt TJ, Sande C, Srikantiah P, Thacker N, Waldstein KA, Weinberger D, Wildenbeest J, Wiseman D, Zar HJ, Zambon M, Bont L. 2023. Respiratory syncytial virus prevention within reach: the vaccine and monoclonal antibody landscape. Lancet Infect Dis 23:e2–e21. doi:10.1016/S1473-3099(22)00291-2. 35952703 PMC9896921

[19] Pfizer. 25 August 2022. Pfizer announces positive top-line data from phase 3 trial of older adults for its bivalent respiratory syncytial virus (RSV) vaccine candidate. https://www.pfizer.com/news/press-release/press-release-detail/pfizer-announces-positive-top-line-data-phase-3-trial-older.

[20] Pfizer. 1 November 2022. Pfizer announces positive top-line data of phase 3 global maternal immunization trial for its bivalent respiratory syncytial virus (RSV) vaccine candidate. https://www.pfizer.com/news/press-release/press-release-detail/pfizer-announces-positive-top-line-data-phase-3-global.

[21] GSK. 13 October 2022. GSK’s older adult respiratory syncytial virus (RSV) vaccine candidate shows 94.1% reduction in severe RSV disease and overall vaccine efficacy of 82.6% in pivotal trial. https://www.gsk.com/en-gb/media/press-releases/gsk-s-older-adult-respiratory-syncytial-virus-rsv-vaccine-candidate/.

[22] Johnson and Johnson. 29 September 2021. Janssen announces start of phase 3 trial for investigational respiratory syncytial virus (RSV) vaccine in older adults. https://www.jnj.com/janssen-announces-start-of-phase-3-trial-for-investigational-respiratory-syncytial-virus-rsv-vaccine-in-older-adults.

[23] Staff. 11 June 2022. mRNA-1345 respiratory syncytial virus (RSV) vaccine. https://www.precisionvaccinations.com/vaccines/mrna-1345-respiratory-syncytial-virus-rsv-vaccine.

[24] Boukhvalova MS, Prince GA, Blanco JC. 2010. Inactivation of respiratory syncytial virus by zinc finger reactive compounds. Virol J 7:20. doi:10.1186/1743-422X-7-20. 20102602 PMC2823672

[25] Shafique M, Wilschut J, de Haan A. 2012. Induction of mucosal and systemic immunity against respiratory syncytial virus by inactivated virus supplemented with TLR9 and NOD2 ligands. Vaccine 30:597–606. doi:10.1016/j.vaccine.2011.11.054. 22120195

[26] Zhao Y, Ma C, Yang J, Zou X, Pan Z. 2021. Dynamic host immune and transcriptomic responses to respiratory syncytial virus infection in a vaccination-challenge mouse model. Virol Sin 36:1327–1340. doi:10.1007/s12250-021-00418-3. 34138405 PMC8692543

[27] Seo HS. 2015. Application of radiation technology in vaccines development. Clin Exp Vaccine Res 4:145–158. doi:10.7774/cevr.2015.4.2.145. 26273573 PMC4524899

[28] Bayer L, Fertey J, Ulbert S, Grunwald T. 2018. Immunization with an adjuvanted low-energy electron irradiation inactivated respiratory syncytial virus vaccine shows immunoprotective activity in mice. Vaccine 36:1561–1569. doi:10.1016/j.vaccine.2018.02.014. 29439869

[29] Ji HJ, Byun EB, Chen F, Ahn KB, Jung HK, Han SH, Lim JH, Won Y, Moon JY, Hur J, Seo HS. 2021. Radiation-inactivated S. gallinarum vaccine provides a high protective immune response by activating both humoral and cellular immunity. Front Immunol 12:717556. doi:10.3389/fimmu.2021.717556. 34484221 PMC8415480

[30] Furuya Y, Regner M, Lobigs M, Koskinen A, Mullbacher A, Alsharifi M. 2010. Effect of inactivation method on the cross-protective immunity induced by whole ‘killed’ influenza A viruses and commercial vaccine preparations. J Gen Virol 91:1450–1460. doi:10.1099/vir.0.018168-0. 20147516

[31] Singleton EV, David SC, Davies JB, Hirst TR, Paton JC, Beard MR, Hemmatzadeh F, Alsharifi M. 2020. Sterility of gamma-irradiated pathogens: a new mathematical formula to calculate sterilizing doses. J Radiat Res 61:886–894. doi:10.1093/jrr/rraa076. 32930781 PMC7674690

[32] Vekemans J, Moorthy V, Giersing B, Friede M, Hombach J, Arora N, Modjarrad K, Smith PG, Karron R, Graham B, Kaslow DC. 2019. Respiratory syncytial virus vaccine research and development: World Health Organization technological roadmap and preferred product characteristics. Vaccine 37:7394–7395. doi:10.1016/j.vaccine.2017.09.092. 29395536

[33] McLellan JS, Yang Y, Graham BS, Kwong PD. 2011. Structure of respiratory syncytial virus fusion glycoprotein in the postfusion conformation reveals preservation of neutralizing epitopes. J Virol 85:7788–7796. doi:10.1128/JVI.00555-11. 21613394 PMC3147929

[34] Schneider-Ohrum K, Cayatte C, Bennett AS, Rajani GM, McTamney P, Nacel K, Hostetler L, Cheng L, Ren K, O’Day T, Prince GA, McCarthy MP. 2017. Immunization with low doses of recombinant postfusion or prefusion respiratory syncytial virus F primes for vaccine-enhanced disease in the cotton rat model independently of the presence of a Th1-biasing (GLA-SE) or Th2-biasing (alum) adjuvant. J Virol 91:e02180-16. doi:10.1128/JVI.02180-16. 28148790 PMC5375676

[35] Thaysen-Andersen M, Jorgensen SB, Wilhelmsen ES, Petersen JW, Hojrup P. 2007. Investigation of the detoxification mechanism of formaldehyde-treated tetanus toxin. Vaccine 25:2213–2227. doi:10.1016/j.vaccine.2006.12.033. 17240009

[36] Techaarpornkul S, Barretto N, Peeples ME. 2001. Functional analysis of recombinant respiratory syncytial virus deletion mutants lacking the small hydrophobic and/or attachment glycoprotein gene. J Virol 75:6825–6834. doi:10.1128/JVI.75.15.6825-6834.2001. 11435561 PMC114409

[37] Shahriari S, Wei KJ, Ghildyal R. 2018. Respiratory syncytial virus matrix (M) protein interacts with actin in vitro and in cell culture. Viruses 10:535. doi:10.3390/v10100535. 30274351 PMC6213044

[38] Battles MB, McLellan JS. 2019. Respiratory syncytial virus entry and how to block it. Nat Rev Microbiol 17:233–245. doi:10.1038/s41579-019-0149-x. 30723301 PMC7096974

[39] Flynn JA, Durr E, Swoyer R, Cejas PJ, Horton MS, Galli JD, Cosmi SA, Espeseth AS, Bett AJ, Zhang L. 2016. Stability characterization of a vaccine antigen based on the respiratory syncytial virus fusion glycoprotein. PLoS One 11:e0164789. doi:10.1371/journal.pone.0164789. 27764150 PMC5072732

[40] Graham BS. 2017. Vaccine development for respiratory syncytial virus. Curr Opin Virol 23:107–112. doi:10.1016/j.coviro.2017.03.012. 28525878 PMC5653266

[41] Connors M, Giese NA, Kulkarni AB, Firestone CY, Morse HC, III, Murphy BR. 1994. Enhanced pulmonary histopathology induced by respiratory syncytial virus (RSV) challenge of formalin-inactivated RSV-immunized BALB/c mice is abrogated by depletion of interleukin-4 (IL-4) and IL-10. J Virol 68:5321–5325. doi:10.1128/JVI.68.8.5321-5325.1994. 8035532 PMC236482

[42] Waris ME, Tsou C, Erdman DD, Zaki SR, Anderson LJ. 1996. Respiratory syncytial virus infection in BALB/c mice previously immunized with formalin-inactivated virus induces enhanced pulmonary inflammatory response with a predominant Th2-like cytokine pattern. J Virol 70:2852–2860. doi:10.1128/JVI.70.5.2852-2860.1996. 8627759 PMC190142

[43] Smith G, Raghunandan R, Wu Y, Liu Y, Massare M, Nathan M, Zhou B, Lu H, Boddapati S, Li J, Flyer D, Glenn G. 2012. Respiratory syncytial virus fusion glycoprotein expressed in insect cells form protein nanoparticles that induce protective immunity in cotton rats. PLoS One 7:e50852. doi:10.1371/journal.pone.0050852. 23226404 PMC3511306

[44] McKimm-Breschkin JL. 2004. A simplified plaque assay for respiratory syncytial virus—direct visualization of plaques without immunostaining. J Virol Methods 120:113–117. doi:10.1016/j.jviromet.2004.02.020. 15234816

[45] Holly J, Fogelova M, Jakubcova L, Tomcikova K, Vozarova M, Vareckova E, Kostolansky F. 2017. Comparison of infectious influenza A virus quantification methods employing immuno-staining. J Virol Methods 247:107–113. doi:10.1016/j.jviromet.2017.06.004. 28610903

[46] Reed LJ, Muench H. 1938. A simple method of estimating fifty per cent endpoints. Am J Epidemiol 27:493–497. doi:10.1093/oxfordjournals.aje.a118408.

[47] Kim KH, Lee YT, Hwang HS, Kwon YM, Kim MC, Ko EJ, Lee JS, Lee Y, Kang SM. 2015. Virus-like particle vaccine containing the F protein of respiratory syncytial virus confers protection without pulmonary disease by modulating specific subsets of dendritic cells and effector T cells. J Virol 89:11692–11705. doi:10.1128/JVI.02018-15. 26355098 PMC4645681

