# Supplementary figures and images for: Gamma Irradiation-Inactivated Respiratory Syncytial Virus Vaccine Provides Protection but Exacerbates Pulmonary Inflammation by Switching from Prefusion to Postfusion F Protein
Source: Microbiol Spectr. 2023 Jun 5;11(4):e01358-23. doi: 10.1128/spectrum.01358-23 (PMC10434263; doi:10.1128/spectrum.01358-23)

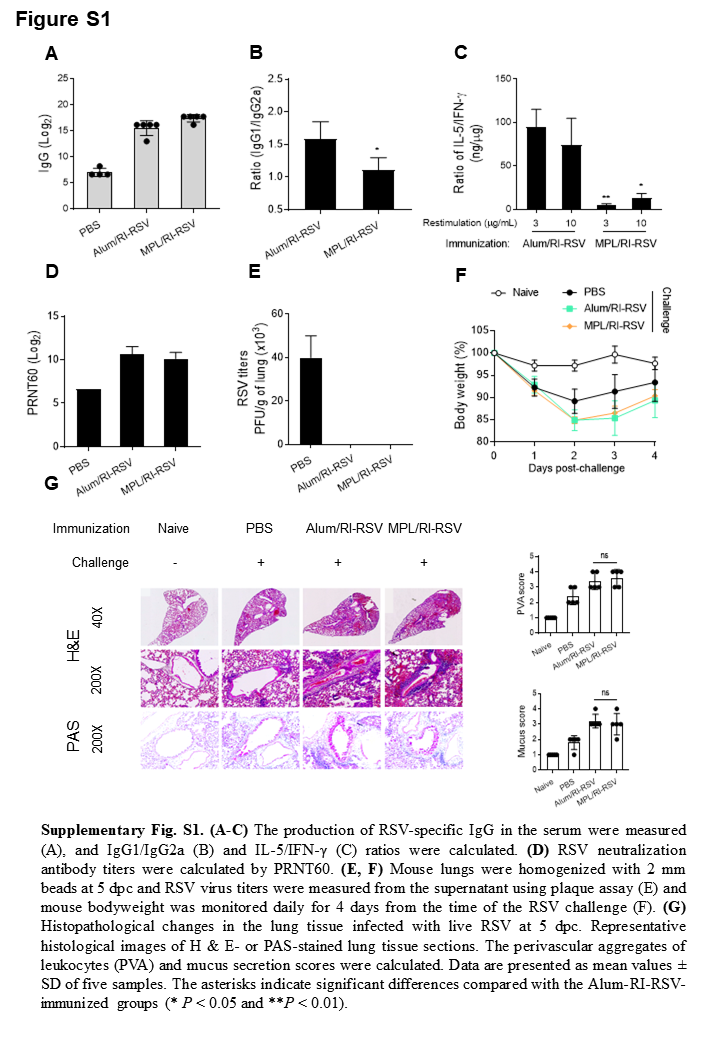

Supplement: Supplemental file 1 — Supplemental material. Download spectrum.01358-23-s0001.tif, TIF file, 0.3 MB [file spectrum.01358-23-s0001.tif]
